# Association of *ST6GAL1* and *CYP19A1* polymorphisms in the 3′-UTR with astrocytoma risk and prognosis in a Chinese Han population

**DOI:** 10.1186/s12885-021-08110-1

**Published:** 2021-04-09

**Authors:** Tuo Wang, Yao Sun, Zichao Xiong, Jiamin Wu, Xiaoying Ding, Xiaoye Guo, Yuan Shao

**Affiliations:** 1 Department of Neurosurgery, the First Affiliated Hospital of Xi’an Jiaotong University, Xi’an, 710061 China; 2 Key Laboratory of Resource Biology and Biotechnology in Western China, Ministry of Education, School of Medicine, Northwest University, Xi’an, 710069 China; 3 Department of Otorhinolaryngology Head and Neck Surgery, the First Affiliated Hospital of Xi’an Jiaotong University, 277 YanTa West Road, Xi’an, 710061 Shaanxi China

**Keywords:** Astrocytoma, *SY6GAL1* gene, *CYP19A1* gene, Risk, Prognosis

## Abstract

**Background:**

Astrocytoma is a common type of central nervous system tumor. In this study, we investigated the correlation between *ST6GAL1* and *CYP19A1* polymorphisms and the risk and prognosis of astrocytoma.

**Methods:**

A total of 365 astrocytoma patients and 379 healthy controls were genotyped using the Agena MassARRAY system. The correlation between *ST6GAL1* and *CYP19A1* variants and astrocytoma risk was calculated using logistic regression. The survival rate of patients with astrocytoma was analyzed to evaluate prognosis.

**Results:**

We found that the *ST6GAL1*-rs2239611 significantly decreased the risk of astrocytoma in the codominant model (*p* = 0.044) and dominant model (*p* = 0.049). In stratified analyses, *CYP19A1*-rs2255192 might be associated with a higher risk of astrocytoma among the low-grade subgroup under recessive (*p* = 0.034) and additive (*p* = 0.030) models. However, *CYP19A1*-rs4646 had a risk-decreasing effect on the high-grade subgroup in the codominant model (*p* = 0.044). The results of Cox regression analysis showed that the CYP19A1-rs2239611 and -rs1042757 polymorphisms were significantly correlated with the prognosis of astrocytoma.

**Conclusion:**

Our results suggest that *ST6GAL1* and *CYP19A1* genes may be a potential biomarker of genetic susceptibility and prognosis to astrocytoma in the Chinese Han population.

**Supplementary Information:**

The online version contains supplementary material available at 10.1186/s12885-021-08110-1.

## Background

Astrocytoma is a malignant tumor that is common and difficult to treat in the central nervous system. It is often fatal because many drugs that are effective against tumors throughout the body cannot cross the blood-brain barrier. Despite advances in diagnostic and therapeutic strategies, patients with astrocytoma have poor survival rates and unfavorable prognosis. The etiology of astrocytoma involves various respects. For example, environmental factors, such as ionizing radiation, some toxic agents, air pollution, and radiofrequency electromagnetic waves, are associated with an increased risk of astrocytoma [1]. Besides, recent studies demonstrated that the role of genetic polymorphisms in the susceptibility and prognosis of astrocytoma has aroused great concern. Single nucleotide polymorphisms of some genes have been shown to be associated with the risk or prognosis of astrocytoma, such as *AKAP6*, *MGMT, EGFR, TERT* [2–5].

ST6 beta-galactoside alpha-2, 6-sialyltransferase 1 (*ST6GAL1*) gene encodes a member of glycosyltransferase family 29. This enzyme plays a vital role in physiological processes such as cell adhesion, antigen recognition, and signal transduction. *ST6GAL1* catalyzes the transfer of sialic acid residues (Neu5Ac) to cell membrane glycoproteins or glycoprotein termini using sialic acid (CMP-Neu5Ac) as the substrate [6]. Increasing evidence has shown that *ST6GAL1* expression and activity changes are often closely related to tumor proliferation, migration, and invasion. Lin et al. [7] hypothesized that down-regulation of *ST6GAL1* expression inhibits the metastasis of breast cancer cells. Zhang et al. [8] found that *ST6GAL1* expression was significantly increased in colorectal cancer. Kroes et al. [9] demonstrated that epigenetic modulation of *ST6GAL1* expression plays a key role in the glioma phenotype in vitro. These results suggested that *ST6GAL1* played a crucial role in cancer development. However, no literature supports the effect of *ST6GAL1*polymorphisms on astrocytoma.

Cytochrome P450 family 19 subfamily A member 1 (*CYP19A1*) encodes aromatase which plays an important role in androgen metabolism [10]. Moreover, the androgen receptor can regulate the proliferation and survival of tumor cells by participating in various oncogenic pathways such as the *MAPK* signaling pathway. Previous studies have shown that sex hormones and their receptors play an important role in the development of many malignant tumors, such as breast cancer, prostate cancer, and colorectal cancer [11–13]. Therefore, we speculated that *CYP19A1* may be involved in the occurrence of cancer by regulating the expression of sex steroid hormones. In addition, the result of bioinformatics analysis implied that *CYP19A1* gene expression was up-regulated in glioblastoma [14]. This evidence led us to think that the *CYP19A1* gene may be involved in the development of astrocytoma.

In this case-control study, we mainly investigated the role of *ST6GAL1* and *CYP19A1* polymorphisms in the occurrence of astrocytoma. Therefore, we selected four polymorphic sites of *ST6GAL1* and *CYP19A1* 3’UTR to explore their impact on the risk and prognosis of astrocytoma.

## Methods

### Informed consent

This study was approved by the ethics committee of the First Affiliated Hospital of Xi’an Jiaotong University, and the experimental protocol was in accordance with the Declaration of Helsinki. All participants signed informed consent forms before participating in this study.

### Study participants

From March 2013 to December 2017, we recruited 365 astrocytomas (age 42.87 ± 17.43 years) and 379 controls (age 45.01 ± 7.08 years) from the Tangdu Hospital and the First Affiliated Hospital of Xi’an Jiaotong University. All included patients had recently diagnosed and histopathologically confirmed astrocytoma according to the World Health Organization (WHO) classification in 2007. Patients with any history of other cancers, having undergone radiotherapy, chemotherapy, or surgery and inflammatory diseases were excluded. Demographic and clinical information was gathered from medical records, questionnaires, and follow-up. These data included age, gender, diagnosed date of the primary tumor, the extent of surgery, radiation therapy, and/ or chemotherapy, the last follow-up date, and status of patient at the time of last follow-up. The inclusion criteria of the control group included no medical or family history of cancer or any neurogenic diseases. At the time of recruitment, each subject was personally interviewed by trained personnel using a structured questionnaire to obtain information regarding demographic characteristics.

Subsequently, patients diagnosed with astrocytoma were followed up by personal or family contacts from the time of diagnosis until death or the last follow-up. With the approval of the patients and their families, follow-up was carried out regularly by telephone, outpatient visits, letters, or clinical data consulting. The patients were followed up every 3 months for 48 months, and the follow-up deadline of this study was December 2017. Follow-up included gathering information regarding treatment (whether to undergo surgery, whether to receive radiotherapy or chemotherapy) and survival status (death, date of death). The patients’ condition changes were determined regularly through follow-up feedback, and clinical pathology and survival rate data were collected for statistical analysis.

### SNP genotyping

We identified two SNPs (rs2239611, rs1042757) in *ST6GAL1* and two SNPs (rs2255192, rs4646) in *CYP19A1* with a minor allele frequency (MAF) ≥ 5% of the 1000 Genomes Project Data. The DNA was extracted from peripheral blood using the DNA purification kit (GoldMag Co. Ltd., Xi’an, China). The concentration and purity of DNA were measured using the NanoDrop 2000 (Thermo Scientific, Waltham, MA, USA). Genotyping of *ST6GAL1* and *CYP19A1* were performed by the Agena MassARRAY platform (Agena Bioscience, San Diego, CA, USA) as described in previous studies [15]. MassARRAY Typer 4.0 software was used for data management and analysis.

### Statistical analyses

Statistical analyses were performed using SPSS software (version 21.0, IBM Corporation, Armonk, NY, USA) and PLINK (http://zzz.bwh.harvard.edu/plink/ld.shtml). We conducted Pearson’s χ^2^ test and student’s *t*-test to assess differences in age and gender between cases and controls. Deviation from Hardy-Weinberg equilibrium (HWE) was evaluated using the chi-square test [16]. Logistic regression was used to calculate odds ratios (OR) and 95% confidence interval (CI) to evaluate the relationship between polymorphic loci and genetic susceptibility to astrocytoma [17]. The Kaplan-Meier method and Log-rank test were used to plot survival curves. Univariate and multivariate Cox regression was used to calculate the Hazard Ratios (HR) and 95% CI. *p* <  0.05 was considered statistically significant.

## Results

### Characteristics of subjects

Table 1 showed clinical and follow-up information for the 365 patients with astrocytoma. There was a significant difference in the age distribution (*p* <  0.001) between the two groups, but not found in gender (*p* = 0.725). In the present study, 89% of the patients received some type of surgical treatment (gross total resection (GTR), near-total resection (NTR), and sub-total resection (STR)), and 40% of patients received chemotherapy after surgery. Moreover, we found that surgical treatment and chemotherapy had a significant impact on the survival rate (*p* <  0.001).

**Table 1:** Relationship between clinicopathologic parameters and overall and progression-free survival of 365 astrocytoma patients

Variables		Frequency	Percent	Overall survival (OS)			Progression-free survival (PFS)		
				χ^2^	Log-Rank *p*	1-year OS rate	χ^2^	Log-Rank *p*	1/2/3-year PFS rate
Sex	Male	205	56.2	1.720	0.190	33.70%	2.923	0.087	21.1%/12.6%/8.3%
	Female	160	43.8			33.80%			11.3%/9.3%/7.6%
Age	< 40	155	42.5	3.279	0.070	33.90%	3.351	0.067	18.8%/16.1%/12.7%
	≥40	210	57.5			33.10%			15.4%/6.6%/4.7%
WHO grade	I	36	9.9	1.956	0.376	30.60%	2.267	0.322	22.2%/16.7%/−
	II	202	55.3			33.20%			17.3%/10.8%/9.8%
	III	127	34.8			29.10%			14.5%/8.9%/4.4%
Surgical method	GTR	248	68.0	39.958	**< 0.001**	36.30%	170.895	**< 0.001**	24.1%/15.0%/11.3%
	NTR	114	31.2			21.90%			1.8%/−/−
	STR	3	0.8			0.00%			0/−/−
Radiotherapy	No	40	11.0	1.213	0.545	45.00%	4.962	0.084	17.5%/−/−
	Gamma knife	222	60.8			33.80%			16.3%/7.1%/6.4%
	Conformal radiotherapy	103	28.2			21.40%			17.8%/16.7%/10.6%
Chemotherapy	No	219	60.0	25.403	**< 0.001**	26.90%	15.193	**0.002**	16.0%/6.8%/5.1%
	Platinum-based	75	20.5			26.70%			12.0%/−/4.0%
	ACNU	41	11.3			43.90%			10.0%/−/−
	TMZ	30	8.2			60.00%			45.6%/41.5%/−
Status	Survival	22	6.0						
	Missing	15	4.1						
	Death	328	89.9						

Log-rank *p* values were calculated using the Chi-Square test

Bold values indicate a significant difference (*p* < 0.05)

*GTR* Gross total resection, *NTR* Near-total resection, *STR* Sub-total resection, *ACNU* Nimustine, *TMZ* Temozolomide

### Association between the *ST6GAL1*/*CYP19A1* genes and astrocytoma risk

Four SNPs (rs2239611, rs1042757, rs2255192, rs4646) were genotyped in this study. The basic information for these SNPs was shown in Supplementary Table 1. The genotype distribution of all SNPs in control was in accordance with HWE (*p* > 0.05). We also observed that these SNPs were located in the 3′-UTR region of *ST6GAL1* and *CYP19A1.*

Table 2 showed that rs2239611 of *ST6GAL1* gene significantly decreased the risk of astrocytoma in the codominant model (OR = 0.87, 95% CI = 0.41–1.83, *p* = 0.044) and the dominant model (OR = 0.74, 95% CI = 0.55–1.00, *p* = 0.049) after adjusted by age and gender. No significant difference was found for the other SNPs between cases and controls (all *p* > 0.05, not shown).

**Table 2:** Genotypic model analysis of the relationship between SNPs and risk of astrocytoma

Gene	SNP ID	Model	Genotype	Controls	Cases	Crude		Adjusted by gender and age	
						OR (95% CI)	*p*	OR (95% CI)	*p*
*ST6GAL1*	rs2239611	Codominant	G/G	225 (59.4%)	244 (66.8%)	1.00	**0.034**	1.00	**0.044**
			A/G	138 (36.4%)	107 (29.4%)	**0.81 (0.39–1.69)**		**0.87 (0.41–1.83)**	
			A/A	16 (4.2%)	14 (3.8%)	0.71 (0.52–0.98)		0.72 (0.53–0.99)	
		Dominant	G/G	225 (59.4%)	244 (66.8)	1.00	**0.035**	1.00	**0.049**
			A/G-A/A	154 (40.6)	121 (33.2%)	**0.72 (0.54–0.98)**		**0.74 (0.55–1.00)**	
		Recessive	G/G-A/G	363 (95.8%)	351 (96.2%)	1.00	0.789	1.00	0.932
			A/A	16 (4.2%)	14 (3.8%)	0.90 (0.44–1.88)		0.97 (0.46–2.02)	
		Additive	–	–	–	0.78 (0.61–1.01)	0.059	0.80 (0.62–1.04)	0.089

*p* values were calculated by logistic regression analysis with adjustments for age and gender

Bold values indicate a significant difference (*p* < 0.05)

*SNP* Single nucleotide polymorphism, *OR* Odds ratio, *95% CI* 95% confidence interval

### Stratification analysis by gender and WHO grade

Next, we conducted stratified analysis by gender and WHO grade, as presented in Table 3. Our results showed that rs2255192 in the *CYP19A1* gene significantly increased the risk of astrocytoma among the low-grade subgroup under the recessive model (OR = 3.05, 95% CI = 1.11–8.34, *p* = 0.034) and the additive model (OR = 1.53, 95% CI = 1.04–2.24, *p* = 0.03). In addition, *CYP19A1-*rs4646 had a risk- decreasing effect on high-grade subgroup under the codominant model (OR = 0.61, 95% CI = 0.39–0.95, *p* = 0.044). However, the results of the gender stratification analysis revealed no significant association between the SNPs and the risk of astrocytoma (all *p* > 0.05, not shown).

**Table 3:** Association of SNPs and astrocytoma risk stratified by WHO grade

Gene	SNP ID	Model	Genotype	LGA				HGA			
				Controls	Cases	OR (95% CI)	*p*	Controls	Cases	OR (95% CI)	*p*
*CYP19A1*	rs2255192	Codominant	C/C	257 (68.0%)	150 (63.6%)	1.00	0.058	257 (68.0%)	83 (65.3%)	1.00	0.800
			C/T	109 (28.8%)	71 (30.1%)	1.31 (0.81–2.13)		109 (28.8%)	41 (32.3%)	1.11 (0.71–1.73)	
			T/T	12 (3.2%)	15 (6.4%)	3.33 (1.20–9.23)		12 (3.2%)	3 (2.4%)	0.76 (0.20–2.82)	
		Dominant	C/C	257 (68.0%)	150 (63.6%)	1.00	0.096	257 (68.0%)	83 (65.3%)	1.00	0.740
			C/T-T/T	121 (32.0%)	86 (36.4%)	1.48 (0.93–2.35)		121 (32.0%)	44 (34.6%)	1.08 (0.70–1.66)	
		Recessive	C/C-C/T	366 (96.8%)	221 (93.6%)	1.00	**0.034**	366 (96.8%)	124 (97.6%)	1.00	0.630
			T/T	12 (3.2%)	15 (6.4%)	**3.05 (1.11–8.34)**		12 (3.2%)	3 (2.4%)	0.73 (0.20–2.71)	
		Additive	–	–	–	**1.53 (1.04–2.24)**	**0.030**	–	–	1.03 (0.71–1.50)	0.880
	rs4646	Codominant	C/C	188 (49.7%)	120 (50.6%)	1.00	0.420	188 (49.7%)	74 (58.3%)	1.00	**0.044**
			C/A	163 (43.1%)	103 (43.5%)	0.76 (0.48–1.20)		163 (43.1%)	40 (31.5%)	**0.61 (0.39–0.95)**	
			A/A	27 (7.1%)	14 (5.9%)	0.66 (0.24–1.80)		27 (7.1%)	13 (10.2%)	1.24 (0.60–2.55)	
		Dominant	C/C	188 (49.7%)	120 (50.6%)	1.00	0.200	188 (49.7%)	74 (58.3%)	1.00	0.083
			C/A-A/A	190 (50.3%)	117 (49.4%)	0.75 (0.48–1.17)		190 (50.3%)	53 (41.7%)	0.69 (0.46–1.05)	
		Recessive	C/C-C/A	351 (92.9%)	223 (94.1%)	1.00	0.570	351 (92.9%)	114 (89.8%)	1.00	0.250
			A/A	27 (7.1%)	14 (5.9%)	0.76 (0.28–2.01)		27 (7.1%)	13 (10.2%)	1.52 (0.75–3.07)	
		Additive	–	–	–	0.78 (0.53–1.14)	0.190	–	–	0.87 (0.62–1.20)	0.390

*p* values were calculated by logistic regression analysis with adjustments for age and gender

Bold values indicate a significant difference (*p* < 0.05)

*LGA* Low-grade astrocytoma, *HGA* High-grade astrocytoma, *OR* Odds ratio, *95% CI* 95% confidence interval

### Association between clinic pathologic factors and genotypes and survival

**Fig. 1:**
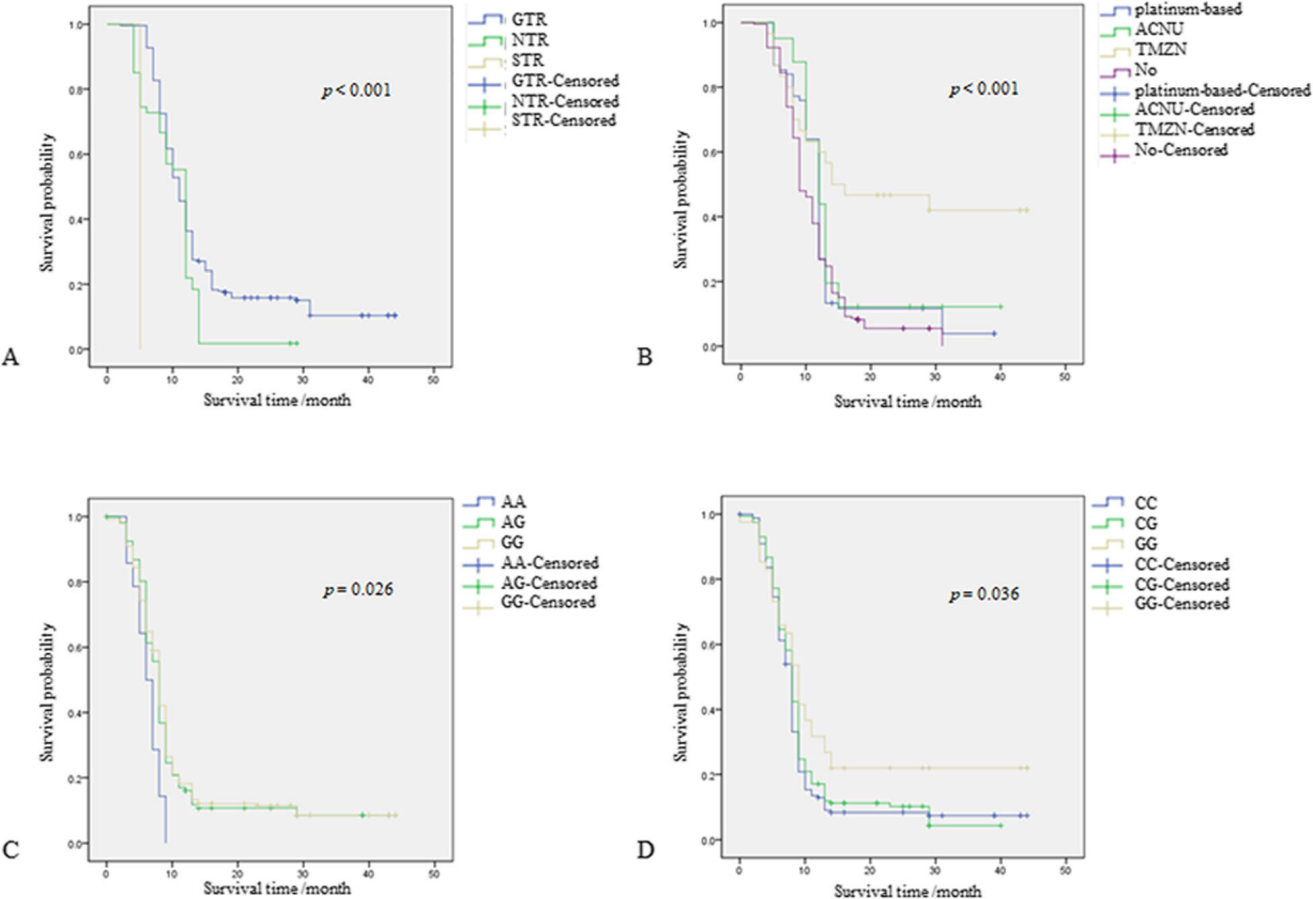
Correlation between clinical information and genotype and astrocytoma prognosis. **a** Surgical method is significantly related to astrocytoma prognosis. **b** Chemotherapy is significantly related to astrocytoma prognosis. **c** *ST6GAL1* rs2239611 is significantly related to astrocytoma prognosis. **d** *ST6GAL1* rs1042757 is significantly related to astrocytoma prognosis

The log-rank test was used to determine the correlation between clinical information and genotype and the prognosis of astrocytoma. The association between genetic polymorphisms and astrocytoma prognosis was determined by Kaplan-Meier survival analysis. As shown in Fig. 1, surgical methods, chemotherapy, and genotypes (*ST6GAL1*-rs2239611, *−*rs1042757) were the primary prognostic factors for astrocytoma (*p* <  0.05). The prognosis of astrocytoma patients undergoing GTR and NTR was better than patients who treated with STR (*p* <  0.001, Fig. 1a). Chemotherapy (platinum-based, ACNU, TMZ) had a positive effect on the prognosis of patients with astrocytoma (*p* <  0.001, Fig. 1b). Besides, *ST6GAL1*-rs2239611 AG or GG genotype was significantly associated with improved OS of astrocytoma (*p* = 0.026, Fig. 1c). We observed an increased survival for *ST6GAL1*-rs1042757 GG genotype (*p* = 0.036, Fig. 1d).

Finally, Cox multivariate analysis revealed that GTR, NTR and TMZ were associated with the prognosis of astrocytoma (all *p* <  0.001, Table 4). Patients with GTR (HR = 0.03, 95% CI = 0.01–0.09, *p* <  0.001, for OS; HR = 0.01, 95% CI = 0.00–0.02, *p* <  0.001, for PFS) and NTR (HR = 0.03, 95% CI = 0.01–0.13, *p* <  0.001, for OS; HR = 0.01, 95% CI = 0.00–0.03, *p* < 0.001, for PFS) presented statistically significant association with longer OS and PFS than STR patients. Patients treated with TMZ was significantly associated with improved OS of astrocytoma (HR = 0.29, 95% CI = 0.17–0.50, *p* < 0.001).

**Table 4:** Multivariate analysis of prognostic factors for survival rate in astrocytoma patients

Variable		No. of patients/events	OS		PFS	
			HR (95% CI)	*p*	HR (95% CI)	*p*
Surgical method	STR	3/3	1.00		1.00	
	GTR	248/213	0.03 (0.01–0.09)	**< 0.001**	0.01 (0.00–0.02)	**< 0.001**
	NTR	114/112	0.03 (0.01–0.13)	**< 0.001**	0.01 (0.00–0.03)	**< 0.001**
Chemotherapy	NO	219/205	1.00		1.00	
	Platinum-based	75/70	0.78 (0.59–1.03)	0.075	0.92 (0.69–1.22)	0.544
	ACNU	41/36	0.74 (0.51–1.06)	0.099	0.93 (0.65–1.34)	0.691
	TMZ	30/17	0.29 (0.17–0.50)	**< 0.001**	0.40 (0.23–0.69)	0.395
*ST6GAL1* rs2239611	GG	244/219	1.00		1.00	
	AG	107/95	1.01 (0.79–1.29)	0.947	0.99 (0.76–1.29)	0.945
	AA	14/14	1.92 (1.11–3.34)	**0.020**	1.64 (0.93–2.90)	0.088

Bold values indicate a significant difference (*p* < 0.05)

*OS* Overall survival, *PFS* Progression-free survival, *GTR* Gross total resection, *NTR* Near-total resection, *STR* Sub-total resection, *HR* Hazard ratio, *ACNU* Nimustine, *TMZ* Temozolomide

Besides, for *ST6GAL*- rs2239611, patients with AA genotype (HR = 1.92, 95% CI = 1.11–3.34, *p* = 0.020) had a decreased survival compared with AG and GG patients.

## Discussion

This study explored the correlations between *ST6GAL1* and *CYP19A1* gene polymorphisms and astrocytoma susceptibility and prognosis. Our findings suggest that the *ST6GAL1*-rs2239611 polymorphism is significantly correlated with astrocytoma susceptibility and prognosis. In the stratified analysis, *CYP19A1*-rs2255192 might be associated with a higher risk of astrocytoma among the low-grade subgroup. However, *CYP19A1*-rs4646 had a risk-decreasing effect on the high-grade subgroup. In addition, the results of Cox regression analysis showed that the *ST6GAL1*-rs2239611 and -rs1042757 polymorphisms were significantly correlated with the prognosis of astrocytoma. These data emphasized the crucial role of *ST6GAL1* and *CYP19A1* in the pathogenesis of astrocytoma, and provide new biomarkers for the treatment and diagnosis of astrocytoma.

The *ST6GAL1* gene encodes a glycosyltransferase that is an important member of the ST family. The enzyme plays an important role in the regulation of STs via the synthesis of terminal α-2,6-sialic acid linkages on complex *N*-glycans and in epigenetic modifications of genes [18]. It is reported that *ST6GAL1* is closely related to the formation and progression of a variety of primary tumors [19, 20]. Substantial reports have shown that *ST6GAL1* is overexpressed in various types of cancer, such as oral, ovarian, prostate, hepatic, and glioma [21–24]. Moreover, its expression is positively correlated with the aggressiveness and metastatic potential of tumors. Polymorphisms in *ST6GAL1* may influence the glycosyltransferase function, in turn affecting the function of histones in chromosomal glycosylation [25]. Schultz et al. [26] showed that *ST6GAL1* is upregulated in ovarian and pancreatic carcinomas, and associated with reduced patient survival. It was reported that *ST6GAL1* induces the expression of the key tumor-promoting transcription factors Sox9 and Slug [26]. A previous study also found that high expression of *ST6GAL1* is associated with poor outcomes in melanoma, hepatocellular, breast, and cervical cancers [27]. In our case-control study, we found that *ST6GAL1*-rs2239611 is associated with a decreased risk but the significantly poorer prognosis of astrocytoma. Through database analyses [14], it found that *ST6GAL1* expression is elevated in glioma tissues and cells. Based on these results, we suggested that *ST6GAL1* played a pivotal role in astrocytoma development.

*CYP19A1* encodes aromatase that plays an important role in transcriptional regulation in human carcinogenesis [28, 29]. High *CYP19A1* expression is critical for the development of breast cancer [29]. Friesenhengst et al. [30] reported that the expression of the *CYP19A1* gene plays a key role in determining the malignancy and survival rates in breast cancer. Armamento et al. [31] showed that the *CYP19A1*-rs4646 is associated with disease progression in patients with breast cancer. Moreover, the GTEx Portal database (https://gtexportal.org/home/) revealed that the expression of different rs4646 genotypes in nerve tissue varies significantly (*p* = 6.700 × 10^− 5^). In our study, we observed that *CYP19A1*-rs4646 had a risk-decreasing effect on the high-grade subgroup. Thus, we suggested that *CYP19A1*-rs4646 may be involved in the development and progression of astrocytoma.

We also found that surgical methods and chemotherapy have a significant effect on the prognosis of astrocytoma. Bagante et al. found that surgery and chemotherapy significantly reduce the prognosis risk of patients with biliary tract cancers [32]. Sho et al. demonstrated that adjuvant chemotherapy significantly affects the survival of pancreatic cancer patients after resection [33]. The results of these studies were consistent with our results, showing that surgery and chemotherapy may be important factors in cancer treatment.

Some limitations of this study are as follows. Firstly, we just selected two SNPs of the *ST6GAL1* and *CYP19A1* genes associated with astrocytoma risk, and more SNPs in *ST6GAL1* and *CYP19A1* are needed to detect. Secondly, the molecular mechanism under which *ST6GAL1* and *CYP19A1* polymorphisms affect the risk and prognosis in astrocytoma is not elucidated. Subsequent experiments were used to validate our findings in vivo and in vitro.

## Conclusions

In summary, our study provided evidence that *ST6GAL1* and *CYP19A1* genes contribute to the development of astrocytoma among Chinese Han people. This explorative study might provide valuable insights and serve as the basis for further functional studies. Additionally, it might be effective biomarkers that would aid in the prevention and treatment of astrocytoma. However, the results of this study require validation in other populations and laboratory-based functional studies.

## Supplementary Information

**Additional file 1: Supplementary Table 1.** Basic data regarding the *ST6GAL1* and *CYP19A1* candidate SNPs examined in this study.

## Data Availability

The datasets used and/or analyzed during the current study are available from the corresponding author on reasonable request.

## Abbreviations

HWE: Hardy-Weinberg equilibrium
CYP: Cytochrome P450
MAF: Minor allele frequency
SD: Standard deviation
ORs: Odds ratios
95% CIs: 95% confidence intervals
SNPs: Single-nucleotide polymorphisms
GTR: Gross total resection
NTR: Near-total resection
STR: Sub-total resection
ACNU: Nimustine
TMZ: Temozolomide
OS: Overall survival
PFS: Progression-free survival
HR: Hazard ratio
