# Supplementary material for: Association of ST6GAL1 and CYP19A1 polymorphisms in the 3′-UTR with astrocytoma risk and prognosis in a Chinese Han population
Source: BMC Cancer. 2021 Apr 9;21:391. doi: 10.1186/s12885-021-08110-1 (PMC8034180; doi:10.1186/s12885-021-08110-1)
Supplement: Supplementary file 1 — Additional file 1: Supplementary Table 1. Basic data regarding the ST6GAL1 and CYP19A1 candidate SNPs examined in this study. [file 12885_2021_8110_MOESM1_ESM.docx]

Supplementary Table 1 Basic data regarding the *ST6GAL1* and *CYP19A1* candidate SNPs examined in this study

| Gene | SNP ID | Chr. | Position | Role | Alleles (A/B) | Frequency (MAF) | | *p*-value for HWE | OR (95% CI) | *p* |
| --- | --- | --- | --- | --- | --- | --- | --- | --- | --- | --- |
|  |  |  |  |  |  | Cases | Controls |  |  |  |
| *ST6GAL1* | rs2239611 | 3q27.3 | 186793702 | 3´UTR | A/G | 0.185 | 0.224 | 0.460 | 0.78 (0.61-1.01） | 0.060 |
| *ST6GAL1* | rs1042757 | 3q27.3 | 186796130 | 3´UTR | G/C | 0.329 | 0.300 | 0.807 | 1.14 (0.92-1.42) | 0.237 |
| *CYP19A1* | rs2255192 | 15q21.2 | 51500835 | 3´UTR | T/C | 0.205 | 0.176 | 0.860 | 1.21 (0.93-1.56) | 0.158 |
| *CYP19A1* | rs4646 | 15q21.2 | 51502844 | 3´UTR | A/C | 0.270 | 0.287 | 0.378 | 0.92 (0.73-1.15) | 0.460 |

SNP = single nucleotide polymorphism; A/B = minor/major; MAF = minor allele frequency; HWE = Hardy-Weinberg equilibrium.

*p* < 0.05 indicates statistical significance.
